# TRENDS IN GERIATRIC PHYSICAL ASSAULT INJURIES TREATED IN U.S. EMERGENCY DEPARTMENTS, 2006-2015

**DOI:** 10.1093/geroni/igz038.1784

**Published:** 2019-11-08

**Authors:** Tony Rosen, Sunday Clark, Zach Gassoumis, Jeanine Yonashiro-Cho, Alyssa Elman, David Lachs, Elizabeth Bloemen, Jeffrey Hall

**Affiliations:** 1 Weill Cornell Medical College / NewYork-Presbyterian Hospital, New York, New York, United States; 2 Department of Emergency Medicine, Weill Cornell Medical College / NewYork-Presbyterian Hospital, New York, New York, United States; 3 Leonard Davis School of Gerontology, University of Southern California, Los Angeles, California, United States; 4 Weill Cornell Medical College, New York, New York, United States; 5 University of Colorado School of Medicine, Aurora, Colorado, United States; 6 Centers for Disease Control and Prevention, Atlanta, Georgia, United States

## Abstract

Trends in Geriatric Physical Assault Injuries Treated in US Emergency Departments, 2006-2015 Older adults are common victims of assault, many of which may result in severe injuries. Our objective was to understand temporal and demographic trends in geriatric assault injuries treated at U.S. Emergency Departments (EDs) and to compare these trends to assault injuries in younger adults. We conducted an analysis of assault injuries in patients aged ≥60 compared to patients aged 18-59 treated in EDs during 2006-2015 using the National Electronic Injury Surveillance System-All Injury Program Special Study of Assaults, which collects data from a nationally representative stratified probability sample of U.S. hospitals. Total geriatric assaults seen in EDs increased from 35,135 in 2006 to 69,657 in 2015, a 98% increase. These injuries increased as a percentage of all geriatric injuries treated from 0.9% to 1.1%. Assaults in older men increased 119%, while assaults in older women increased 68%. Among age groups, the biggest percentage increases were among adults aged 60-64 (138%) and aged 65-74 (89%). ED visits for injuries associated with physical elder abuse increased from 13,241 in 2006 to 27,406 in 2015, a 107% increase. During this period, number of younger adults treated for assault did not significantly change. We concluded that geriatric assault injuries, particularly in older men in younger age groups, are dramatically increasing. Further research is needed to better understand these assaults to develop prevention strategies.

